# Potential of Germination in Selected Conditions to Improve the Nutritional and Bioactive Properties of Moringa (*Moringa oleifera* L.)

**DOI:** 10.3390/foods9111639

**Published:** 2020-11-10

**Authors:** Karín E. Coello, Juana Frias, Cristina Martínez-Villaluenga, María Elena Cartea, Rosaura Abilleira, Elena Peñas

**Affiliations:** 1 Escuela Superior Politécnica del Litoral, ESPOL Polytechnic University, Facultad de Ingeniería Mecánica y Ciencias de la Producción, Campus Gustavo Galindo Km 30.5 Vía Perimetral, P.O. Box 09-01-5863 Guayaquil, Ecuador; kcoello@espol.edu.ec; 2 Department of Food Characterization, Quality and Safety, Institute of Food Science, Technology and Nutrition (ICTAN-CSIC), 28006 Madrid, Spain; frias@ictan.csic.es (J.F.); c.m.villaluenga@csic.es (C.M.-V.); 3 Group of Genetics, Breeding and Biochemistry of Brassicas, Biological Mission of Galicia (CSIC), P.O. Box 28, E-36080 Pontevedra, Spain; ecartea@mbg.csic.es (M.E.C.); rabilleira@mbg.csic.es (R.A.)

**Keywords:** moringa, germination, thiamine, riboflavin, phenolic compounds, gamma-aminobutyric acid, glucosinolates, antioxidant activity

## Abstract

*Moringa oleifera* L. is greatly appreciated for its high content of phytochemicals. Although most parts of moringa tree have been widely studied, seeds remained scarcely explored. The first goal of this study was to investigate the effectiveness of germination to improve the nutritional composition (proximate composition and levels of vitamins B1 and B2), content of bioactive compounds (glucosinolates, phenolics and γ-aminobutyric acid, GABA) and antioxidant activity of moringa seed. Germination improved protein, fat, fiber, riboflavin, phenolics, some individual glucosinolates (GLS) and GABA contents, as well as the antioxidant potential in moringa sprouts, but the extent of the improvement depended on germination conditions. The second objective of this work was to identify the optimal germination conditions to maximize nutritional and bioactive quality of moringa by applying multi-response optimization (response surface methodology, RSM). RSM models indicated that 28 °C and 24 h were the optimal conditions to enhance the accumulation of riboflavin, phenolics and antioxidant activity of sprouts, while the highest GABA and total GLS contents were observed at 36 °C for 96 h and thiamine achieved the maximum content at 36 °C for 24 h. These results show that moringa sprouts are promising functional foods that might be also used as ingredients for the elaboration of novel foodstuffs.

## 1. Introduction

Moringa (*Moringa oleifera* L.) is considered a multipurpose crop native of the Himalayan region (India) that was introduced in various parts of the world, such as the Ecuadorian coast, due to its low demand in terms of soil conditions and agronomic practices. Leaves, flowers, stems and roots have been widely studied in moringa and traditionally they have been consumed or used in traditional medicine [1].

Recently, more attention has been paid to its health-promoting properties including antioxidant, hypolipidemic, anti-inflammatory and immunomodulatory properties [2,3]. The high prevalence of non-communicable diseases (NCDs) and the fact that an effective strategy to reduce the scaling of these pathologies in to control the activity of inflammatory mediators via modifiable risk factors such as diet [4], make the consumption of moringa an attractive approach for preventing the development of NCDs. The health-promoting properties of moringa have been attributed to their high content of bioactive compounds, such as phenolic compounds, alkaloids and terpenes, which have been reported to be the most abundant metabolites in moringa leaves [5]. Although moringa leaves have been deeply studied, information on moringa seeds is less abundant. Most of the studies performed in seed have been focused on the evaluation of the proximate composition in defatted moringa seed and oil [6], as well as on the utilization of seeds and isolated seed proteins for animal feeding [7]. Several studies have demonstrated the high nutritional value of moringa seeds due to their content in proteins, polyunsaturated fatty acids, vitamins and minerals [8] and the presence of phytochemicals such as alkaloids (moringine), phenolic compounds, sterols, and glucosinolate derivatives [9,10] suggesting their great potential for development of novel and healthy food products.

Germination is a natural physiological plant process that starts with water uptake and concludes with the appearance of the radicle [11]. This process enhances the nutritional value and phytochemical content in diverse edible seeds [12]. It has been evidenced that germination causes a remarkable increase of the content of group B and C vitamins [13], proteins and the levels of several bioactive compounds such as phenolic compounds and γ-aminobutyric acid (GABA) in grains [14,15]. Biochemical and physiological changes in seeds strongly depends on soaking time and germination conditions (presence/absence of light, temperature and time) [14,16]. Therefore, the careful selection of the most suitable germination conditions for each seed is crucial to improve its nutritional value and health-promoting properties [17]. It has been reported that moringa sprouts obtained at 30 °C for four days exhibited higher lipid (26%) and protein (9.5%) contents than ungerminated seeds [18]. However, to our knowledge, there is no information in the literature regarding the influence of germination conditions on nutritional and phytochemical composition of moringa seeds. The aim of the present study was to investigate the impact of germination temperature and time on the proximate composition and content of thiamine, riboflavin, free phenolic compounds and GABA as well as on the antioxidant activity of moringa sprouts. A multi-response optimization approach was applied to identify the optimal germination conditions that improve nutritional and health-promoting features in sprouted moringa seed. Since naturalness and minimal processing are two of the most desired attributes by consumers nowadays [19], the identification of optimal germination conditions in moringa seeds will help to develop, through an economical technology, novel ingredients from moringa in line with current consumer preferences.

## 2. Materials and Methods

### 2.1. Plant Material

**Figure 1:**
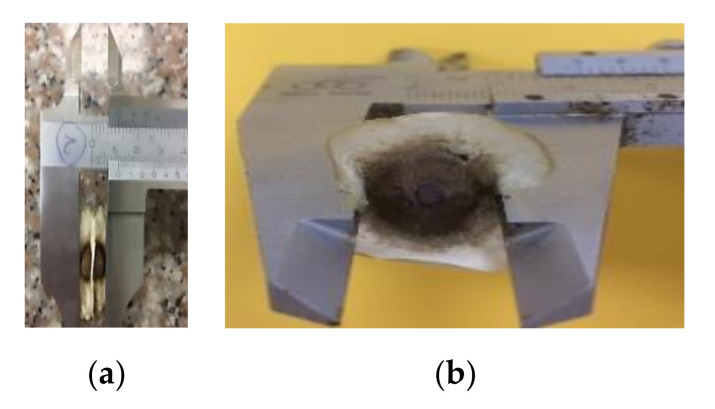
Dimensions of moringa seeds (**a**) width; (**b**) length.

Moringa (*Moringa oleifera* L.) seeds were obtained from trees grown in the Eastern part of Ecuador (Shushufindi) and were kindly provided by a local grower. Seeds were harvested during July 2018 and had an average mass of 0.27 g, average size of 9.17 × 10.44 mm, true density of 0.73 g/ml, bulk density of 0.098 g/ml and porosity of 86.67 (Figure 1). They were stored in vacuum-sealed plastic bags at room temperature and darkness until their use.

### 2.2. Chemicals and Reagents

Unless otherwise specified, all reagents were obtained from Sigma-Aldrich (Barcelona, Spain).

### 2.3. Germination Experiments

Moringa seeds were soaked in 0.1% sodium hypochlorite solution (1:6, *w*/*v*) for 30 min at room temperature and then they were washed with distilled water until they reached neutral pH. Seeds were then soaked with distilled water for 18 h at room temperature (~22 °C). Distilled water was drained, and hydrated seeds were placed in trays on a wet filter paper. Germination was performed in a cabinet (G-120 model, ASL Snijders International S. L., Tilburg, The Netherlands) with an automatic control of temperature and 95% atmospheric humidity. Since germination temperature and time are the most important factors influencing the nutritional quality and the content of bioactive compounds in sprouted seeds [13,14,15,16], different temperatures (28 °C, 32 °C, and 36 °C) and time periods (24, 60, and 96 h) were selected for germination process. Germination temperatures were selected based on the common temperatures used for moringa cultivation in Ecuador, and germination time based on previous studies performed by our group [14]. Moringa sprouts were freeze-dried, milled, packed in plastic bags under vacuum and stored at −20 °C until their use.

### 2.4. Proximate Composition

Nitrogen content was analyzed by the Dumas method, as described in the Association of Official Analytical Chemists AOAC (2000) method 992.23, using a nitrogen analyzer (LECO Corp., St. Joseph, MI, USA). A factor of 5.83 was used for protein content conversion. Fat (AOAC 922.06), ash (AOAC 923.03), and total dietary fiber (AOAC 991.43) were also determined. Total carbohydrates were estimated by difference: 100 − (% proteins + % fat +% ash + % water) [20]. The results were expressed in percentage of dry matter (d.m.).

### 2.5. Thiamine and Riboflavin

A single extraction procedure for vitamins B1 and B2 was carried out. Briefly, 1 g of samples was extracted by acid hydrolysis with 0.3 M HCl (7 mL) in an autoclave for 15 min at 120 °C. After cooling, the pH of samples was adjusted to 5 with 4 M ammonium acetate and then they were incubated with 1.7 mL of a Taka-Diastase solution (20%) for 3 h at 45 °C in agitation. After incubation, sample volume was adjusted to 10 mL with bidistilled water. Extractions were performed in duplicate for each germination condition. Thiamine was quantified by High-Performance Liquid Chromatography (HPLC), using a post-column derivatization chromatographic method, while riboflavin was directly determined by HPLC [21]. Results were expressed in µg/100 g d.m.

### 2.6. Total and Individual GLS

Total GLS (glucosinolates) were extracted from 100 mg of moringa flour samples following a two-step extraction and desulfatation procedure (Kliebenstein et al., 2001) [22]. Extracts were analyzed by Ultra-High-Performance Liquid Chromatography (UHPLC) using an UPLC Nexera LC-30AD chromatograph (Shimadzu, Kyoto, Japan) equipped with a Nexera SIL-30AC injector and SPD-M20A photodiode array detector. The UPLC column was a C18 Atlantis T3 column (3 µm particle size, 2.1 × 100 mm i.d.) from Waters (Waters Corporation, Milford, MA, USA) protected with a C18 guard cartridge. The oven temperature was set at 35 °C. Compounds were eluted at flow rate of 0.8 mL/min by using bidistilled water (mobile phase A) and aqueous acetonitrile (mobile phase B) and the following gradient program: 3 min at 100% H_2_O, 23 min from 0% to 25% acetonitrile, 1 min at 25% (*v*/*v*) acetonitrile, 9 min from 25% to 0% (*v*/*v*) acetonitrile, and 4 min at 100% H_2_O. LabSolutions software Shimadzu (version 5.30 SPI) was used to record the data. Neoglucobrassicin (Phytoplan Diehm and Neuberger GmbH, Heidelberg, Germany) was used as external standard. Results were expressed as µmol/g d.m.

### 2.7. GABA

The extraction and quantification of GABA in moringa seeds and sprouts was performed by Reversed-Phase High-Performance Liquid Chromatography (RP-HPLC). Briefly, 0.5 g of freeze-dried sample was extracted in 10 mL of bidistilled water at 4 °C for 16 h in agitation. After centrifugation (10.000 rpm, 10 °C, 10 min), the supernatant was dried under vacuum conditions and dissolved in 0.5 mL of bidistilled water. Ten µL of the internal standard solution (containing 1.2 mg/mL allyl-L-glycine and 20 μL of 20% *v*/*v* triethylamine in 50% methanol) was added to 50 μL of the extract obtained and the mixture was derivatized by mixing with 30 μL of phenyl isothiocyanate. Then, samples were dried under vacuum conditions, they were dissolved in 0.5 mL of 0.1 M ammonium acetate pH 6.5 and filtered through a 0.22 μm nylon filter for HPLC analysis. HPLC quantification was carried out in an Alliance Separation Module 2695 equipped with a 2996 photodiode array detector (Waters, Milford, KS, USA) using a C18 Alltima column (250 × 4.6 mm, 5 μm, Grace & Co., Albany, NY, USA) at 40 °C. Mobile phases A (0.1 ammonium acetate pH 6.5) and B (0.1 M ammonium acetate, acetonitrile, methanol, 44/46/10, *v*/*v*/*v*, pH 6.5) were used at a flow rate of 0.7 mL/min. The elution gradient was initiated at 100% A for 15 min, then gradient flow from 100% A to 100% B for 27 min, 100% B for 8 min, and finally, 100% A for 5 min. Results were expressed in mg GABA/100 g of dry matter (d.m.).

### 2.8. Free Phenolic Compounds

The content of free phenolic compounds (FPC) was determined in methanolic extracts by the Folin–Ciocalteu’s phenol method, according to Cáceres et al. [14]. Results were expressed as mg of gallic acid equivalents (GAE)/100 g d.m.

### 2.9. Antioxidant Activity

The antioxidant activity was determined in methanolic extracts following the oxygen radical absorbance capacity (ORAC) method previously described by Cáceres et al. [14]. The reaction mixture contained 180 μL of 70 nm fluorescein, 90 μL of 12 mM 2,2′-azo-bis(2-methylpropionamidine) dihydrochloride (AAPH) and 30 μL of diluted sample of the standard (Trolox) at a concentration in the range 1–8 μM. The reaction was performed at 37 °C. The fluorescence was read in a microplate reader (Synergy HT, BioTek Instruments, Winnoski, VT, USA) every minute at λex 485 and λem 520 nm. Results were expressed as mg of Trolox equivalents (TE)/100 g d.m.

### 2.10. Data Modelling and Optimization of Germination Conditions

Response Surface Methodology (RSM) was used to investigate the effect of germination conditions (time and temperature; independent variables) on the content of nutritional and bioactive compounds (response variables) in germinated moringa.

Regression models obtained by response surface methodology (RSM) were used to find out the optimal germination time and temperature for enhancing moringa nutritional and bioactive potential. The response value  y was estimated by the following equation:

(1) $$y=b_{0}+b_{1}x_{1}+b_{2}x_{2}+b_{12}x_{1}x_{2}+b_{11}x_{1}^{2}+b_{22}x_{2}^{2}$$

where $x_{1}$ and $x_{2}$ are the independent variables representing germination time and temperature, respectively; b0 is a constant coefficient; $b_{1}$ and $b_{2}$ are the linear coefficients; and $b_{12}$, $b_{11}$ and $b_{22}$ are the factor interaction coefficients.

The adequacy of the models developed to the experimental data was evaluated by the coefficient of determination (R^2^).

### 2.11. Statistical Analysis

Data shown are the mean values of three experimental replicates of each germination condition ± standard deviation. Data were subjected to one-way analysis of variance (ANOVA) and differences between germination conditions were compared using a Duncan’s multiple-range test with *p* ≤ 0.05 level of significance. The regression models obtained by RSM were validated by multivariate ANOVA. Statgraphics Centurion XVI software, version 16.1.17 (Statistical Graphics Corporation, Rockville, MD, USA) was used for RSM data modelling and ANOVA analyses.

## 3. Results and Discussion

### 3.1. Effect of Germination Conditions on Proximate Composition of Moringa Sprouts

The proximate composition of moringa sprouts is presented in Table 1. Ash content (3.3 g/100 g) in moringa seed is in agreement with literature data [23,24] and it was decreased during germination, suggesting a slight decrease of mineral content in moringa sprouts.

The protein content in moringa seed (~28 g/100 g d.m) was within the range of values earlier reported [25,26]. In general, protein content increased significantly during germination, and the extent of such increment depended on the germination conditions. Moringa sprouts obtained at 32 °C for 60–96 h and at 36 °C for 60 h exhibited the highest protein levels. Ijarotomi et al. [18] reported an enhancement of protein content in moringa germinated at 30 °C for 4 days. The rise in protein levels might be explained by the loss of carbohydrates through respiration, causing perceptual increase in other nutrients, such as proteins. The increase in protein levels observed in moringa sprouted at different germination conditions is less than 15%, results that match with those previously reported in cereal sprouts [27].

Fat content in moringa seed (~29 g/100 g d.m.) (Table 1) was similar to values reported earlier [8,26,28]. In contrast, Olagbemide and Alikwe [24] and Liang et al. [23] reported lower fat levels in moringa seeds than the ones of the present study. Fat content in moringa seed varied depending on germination conditions. Germination at 28 and 36 °C did not result in pronounced modifications of fat content, but temperatures of 32 °C caused a significant (*p* ≤ 0.05) increase in seed fat levels.

Moringa seeds exhibited a total dietary fiber content of 24.4 g/100 g d.m. (Table 1). Dietary fiber increased during germination process and the largest content was found at 32 °C (32–33 g/100 g d.m.). The European Food Safety Authority (EFSA) provide recommendations for consumption of more than 25 g of fiber/day to maintain bowel function, and reduced risk of weight gain, cardiovascular diseases and type 2 diabetes [29]. Taking into account EFSA guidelines, the consumption of 100 g of moringa sprouts per day will satisfy the fiber consumption needs.

Total carbohydrate content in moringa seed was 35.3 g/100 g d.m. (Table 1). During germination, carbohydrate content was reduced. The decrease in carbohydrates levels can be explained by the hydrolysis of starch and the release of reducing sugars that provide energy to the growing seedling [14].

### 3.2. Effect of Germination Conditions on Vitamin B_1_ and B_2_ Contents in Moringa Sprouts

The content of thiamine (vitamin B_1_) and riboflavin (vitamin B_2_) in moringa seeds and sprouts is collected in Table 2. Moringa seeds exhibited levels of thiamine of 1113.5 μg/100 g d.m., which fell within the range of values previously found [25]. Sprouting did not substantially affect the thiamine content in moringa, with exception of germination at 32 °C for 24–96 h and 36 °C for 96 h that showed 10–12% reduction. It has been reported that sprouting stimulates de accumulation of B vitamins in cereal seeds to support seedling development and growth [30]. However, there is no scientific data concerning the effect of germination conditions on thiamine content in moringa seeds. Our results suggest that de novo synthesis of thiamine in moringa is only initiated at lower temperatures in later germination stages, as it has been concluded by Moongngarm and Saetung [31] in sprouted rice, or at high temperatures for short germination times. The leaching of thiamine in water during moringa soaking might explain the slight reduction found in some moringa sprouts.

Riboflavin content in moringa seeds was higher than values previously reported in other moringa varieties [28]. The content of riboflavin in moringa did not change or even increased during germination, and the highest values were observed at 28 °C and 24 h. It has been found that the content of riboflavin increased gradually throughout the germination period up to the fifth day in rapeseeds [32] and wheat seeds [33]. However, our results indicate that riboflavin levels in moringa sprouts decreased with increasing germination time.

### 3.3. Effect of Germination Conditions on Total and Individual GLS Content in Moringa Sprouts

**Figure 2:**
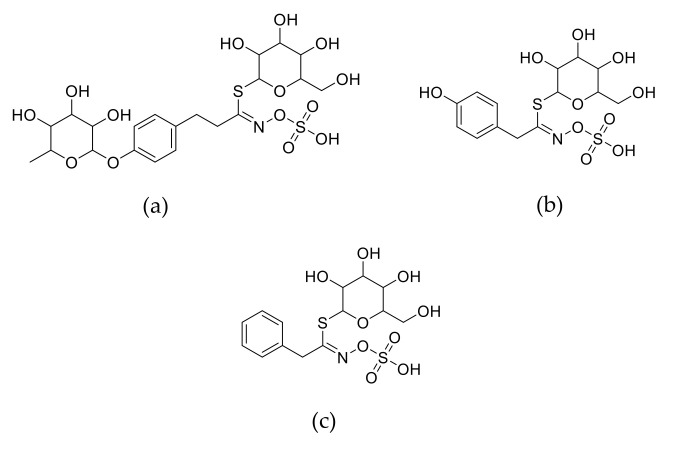
Structure of glucosinolate molecules in moringa sprouts. (**a**) glucomoringin; (**b**) sinalbin; (**c**) glucotropaeolin.

The content of total and individual GLS identified in moringa seeds and sprouts is presented in Table 3. Glucomoringin (4-α-rhamnopyranosyloxy-benzyl GLS) (Figure 2) was the main GLS found in moringa seeds, as reported earlier [9,34,35]. Small amounts of sinalbin (*p*-hydroxybenzyl glucosinolate) (Figure 2, Table 3) were also present in the seed in agreement with a previous study [36].

Significant differences (*p* ≤ 0.05) in the levels of individual GLS between moringa seed and their sprouts were observed. Sprouts exhibited a significant (*p* ≤ 0.05) lower content of sinalbin than seed, whilst changes in glucomoringin depended on germination conditions. Moringa sprouts obtained at 28 °C for 24–60 and 32–36 °C for 96 h presented smaller levels of glucomoringin than seed, while germinated moringa obtained in the rest of germination conditions showed an increase in the content of this compound. Moreover, moringa sprouts obtained in all temperatures at germination periods of 60–96 h also exhibited low amounts of glucotropaeolin (Figure 2), compound that was not found in moringa seeds. These results are consistent with those reported by Maldini et al. [36], who also found small levels of glucotropaeolin in 12-day-old seedlings of moringa but not in seed pulp and coat. Total GLS significantly decreased (*p* ≤ 0.05) in moringa sprouts, with the exception of those produced at 32 °C for 24–60 h and 36°C for 60 h. These results indicate that longer germination periods cause higher diminution of GLS content. As previously reported, postharvest processing strongly affects GLS content in brassica vegetables [37]. Upon cellular injury of plant tissues during processing, GLS are enzymatically degraded by a thioglucosidase enzyme (myrosinase) to a wide variety of breakdown products such as glucose, sulfate, isothiocyanates, epithionitriles, nitriles, indolic alcohols, oxazolidinethions, amines, and thiocyanate [38]. The reduction of total GLS amount observed in most of the moringa sprouts can be attributed to the selective metabolism of some individuals GLS such as sinalbin by myrosinase enzyme [39] during germination. In agreement with our results, recent studies have reported a diminution of total GLS content during sprouting of brassica vegetables such a broccoli and rocket [40,41]. Our results also evidenced that germination in selected conditions activates the biosynthesis of glucomoringin and glucotropaeolin, findings in accordance with those observed by other authors, who reported the increase of levels of some individual GLS in different germinating brassica seeds [42,43]. The branched chain amino transferase, methylthioalkylmalate synthase, and sulfotransferases are key enzymes involved in GLS synthesis, while *BCA*, *CYP* and *AOP* are the most important genes in GLS biosynthetic pathway [44]. Our results suggest that certain germination conditions may influence the expression of these genes, thus affecting the activity of enzymes involved in GLS synthesis pathway.

GLS are sulfur-containing secondary metabolites that alongside with their breakdown products exert chemopreventive, anti-tumor and antimicrobial activity [45]. Current literature has shown that glucomoringin derivatives are able to counteract the inflammatory cascade involved in multiple sclerosis [46] and exert antibacterial activity [47]. Sinalbin has been shown to exhibit in vitro antiproliferative, proapoptotic and antimicrobial effects [48], while glucotropaeolin derivatives showed preventive activity against breast and prostatic cancers in animal models [49,50]. Thus, moringa sprouts can be considered a good source of health-promoting GLS.

### 3.4. Effect of Germination Conditions on GABA Content in Moringa Sprouts

Table 4 summarizes the GABA content in moringa seeds and sprouts obtained at different germination temperatures and times. Non-germinated moringa seeds showed a GABA content of 50.07 mg/100 g d.m., value notably higher than levels reported in other seeds such as pseudocereals [16] and legumes [51]. Germination improved notably the levels of GABA in moringa, showing sprouts increments between 1.8–8.8 fold in the levels of this compound, depending on germination conditions. The only exception was observed when moringa seeds were germinated at 32 °C for 24 h, conditions in which sprouts showed lower GABA concentration than seeds. In general, an increase in GABA was observed with longer germination times, which indicated the strong influence of germination period on its biosynthesis in moringa. The highest GABA accumulation was observed after germination at 36 °C for 96 h (219.19 mg/100 g d.m.).

GABA is a non-protein amino acid that acts as an important depressive neurotransmitter in the nervous system and also as a potent blood pressure and heart rate regulator. Other GABA-related health functions include diabetes prevention, diuresis, and sedation [12]. During germination, endogenous enzyme systems are activated, and proteins are hydrolyzed into free amino acids such as glutamic acid, a GABA precursor. Additionally, glutamate decarboxylase and diamine oxidase, enzymes involved in GABA biosynthesis through decarboxylation of glutamic acid and polyamines, respectively, are activated during germination [50]. The highest GABA content found in moringa sprouts obtained at 36 °C observed in this work is consistent with a previous study showing that the optimal activity of glutamate decarboxylase occurs at temperatures close to 40 °C [52].

### 3.5. Effect of Germination Conditions on the Content of Free Phenolic Compounds (FPC) in Moringa Sprouts

Table 4 collects the FPC in moringa seeds and sprouts produced at different germination conditions. FPC observed in moringa seeds (418.02 mg GAE/100 g d.m.) was higher than that reported for a moringa variety grown in Mexico [53]. Moringa seed is an excellent source of phenolic compounds, exhibiting levels of these phytochemicals much higher than those found in other grains such as cereals, pseudocereals, and other less known sources of sprouts such as teff, evening primrose, phacelia and fenugreek [14,16,54,55].

During germination, changes in phenolic content of moringa strongly depended on experimental conditions. Temperatures ranging 28–32 °C for periods of 24–60 h enhanced the levels of FPC significantly (*p* ≤ 0.05) in moringa. However, 28 °C applied for 96 h and 36 °C for 24–60 h led to lower phenolic contents in the soluble fraction compared to the ungerminated seed. The enhancement of activity and gene expression of key enzymes involved in phenolics biosynthesis during germination have been well documented [56]. The activity of enzymes participating in phenylpropanoid pathway, one of the routes responsible for phenolic accumulation in plants, such as phenylalanine ammonia lyase (PAL), is drastically influenced by germination time and temperature. In this sense, Tesfay et al. [57] observed that the activity of PAL enzyme is maximal at temperatures of 20–30 °C. These findings support the results of the present work, were the highest phenolic content was found at germination temperature of 28 °C for short periods of time. Moreover, activation of cell wall-degrading enzymes (cellulases, endoxylanases and esterases) that hydrolyze phenolic compounds bound to cell wall during germination at temperatures in the range 28–32 °C might also contribute to the increase of phenolic levels in moringa sprouts obtained at these temperatures, as it has been previously reported in other plant sprouts [30].

### 3.6. Effect of Germination Conditions on Antioxidant Activity in Moringa Sprouts

Raw moringa seeds exhibited a high antioxidant potential (1531.36 mg TE/100 g d.m.) (Table 4), which was remarkably enhanced during all germination conditions assayed, with the exception of 36 °C for 96 h. Our results indicate that the accumulation of phenolic compounds in moringa sprouts was positively influenced by mild temperatures and shorter germination times (Table 4), thus contributing to the antioxidant activity of sprouted moringa. It has been shown that germination promotes the antioxidant potential in other plant seeds [16,58]. During germination, there is an uncontrolled production of reactive oxygen species (ROS) that can cause oxidative stress and severe cellular deterioration and, therefore, plant antioxidant enzymes are activated at the early stage of germination [59]. In fact, it has been reported that superoxide dismutase (SOD), an enzyme that plays a crucial role in the major antioxidant defense system against the superoxide radical, is activated during the first day of germination of peanut seed and its activity decrease dramatically afterwards [60]. These findings are in accordance with the highest antioxidant activity of moringa sprouts obtained after 24 h in this work.

Antioxidant activity exhibited a positive correlation with FPC levels, but the low value obtained for the coefficient of correlation (R^2^ = 0.442) suggests that there might be other antioxidant compounds such as vitamins E and C that are also contributing to the antioxidant activity of moringa sprouts.

### 3.7. Multi-Response Modelling of the Influence Germination Conditions on Nutritional and Bioactive Properties of Moringa Sprouts

RSM has been used to better understand the impact of germination time and temperature on nutritional and bioactive profile of germinated moringa. The contribution of each factor to the properties of moringa sprouts is important in order to optimize the conditions of germination process to maximize their quality. Response variables (contents of thiamine, riboflavin, GLS, GABA, FPC and antioxidant activity) were modelled as a function of germination temperature and time. The regression models obtained are presented in Table 5. All models exhibited a non-significant (*p* > 0.05) lack-of-fit and were significant (*p* ≤ 0.05), as evidenced by ANOVA analysis (results not shown). The values of the coefficient of determination higher than 0.75 for all models indicate their suitability to explain the variability of experimental data (Table 5). Both germination time and temperature exerted a strong linear and quadratic influence on the response variables analyzed, being the interactive effect between both factors also significant.

Optimal germination conditions determined by RSM were a temperature of 28 °C for 24 h to enhance the contents of riboflavin, FPC and antioxidant activity in moringa sprouts, while 36 °C in combination with times of 24 and 96 h maximized the levels of thiamine and GABA, respectively (Table 5).

**Figure 3:**
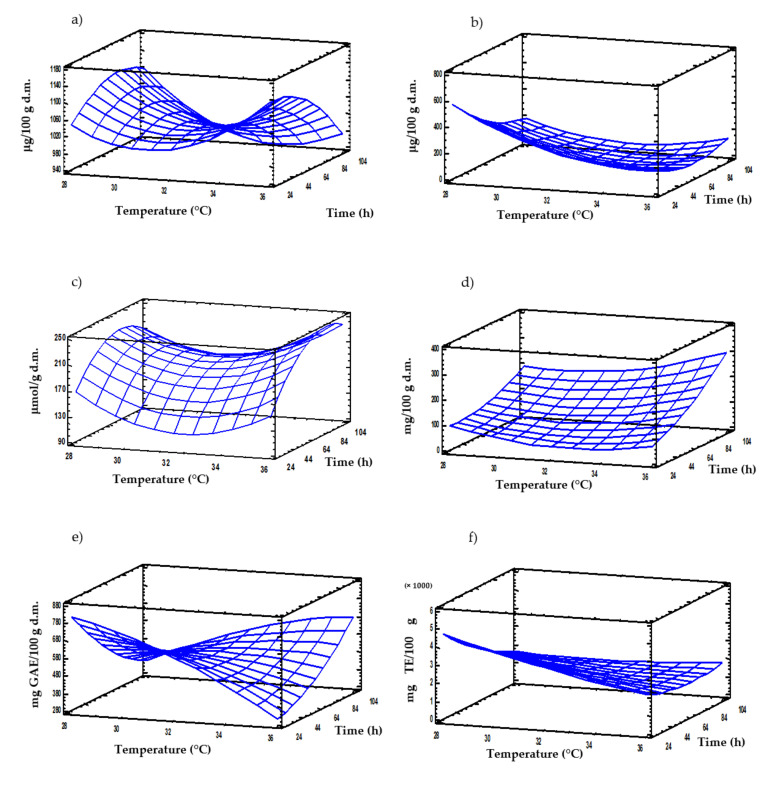
Response surface plots for contents of thiamine (**a**), riboflavin (**b**), glucosinolates (**c**), GABA (**d**), free phenolic compounds (**e**) and antioxidant activity (**f**) in sprouted moringa as a function of germination time and temperature.

Response surface tridimensional plots illustrate the variation of nutritional and bioactive compounds in sprouted moringa as a function of germination temperature and time (Figure 3). Time and temperature exhibited important interactive effects on thiamin content during germination of moringa, and temperature of 36 °C combined with shorter germination times were the conditions that caused the highest accumulation of this vitamin (Figure 3a). Riboflavin content was mainly influenced by germination temperature, while the impact of time was less relevant (Figure 3b). Combinations of low temperatures and short germination times produced moringa sprouts with the largest levels of riboflavin. Germination time was the most important factor influencing the GLS content in moringa sprouts, as previously reported [61], being the effect of temperature less notable (Figure 3c). Short germination times favored the retention of GLS, while longer times caused the reduction of total GLS levels possibly due to the activation of myrosinase enzyme. GABA increased steadily in moringa with germination time, regardless of the temperature used (Figure 3d). Longer germination times and higher temperatures resulted in the most pronounced accumulation of GABA. A decreasing trend was observed for FPC at germination temperatures up to 32 °C with increasing germination time (Figure 3e). However, the opposite behavior occurred at temperatures from 32 to 36 °C, in which FPC content was enhanced as germination time augmented. Germination temperature was the most important factor affecting the antioxidant potential in moringa, being its influence more important at shorter germination times (Figure 3f).

Optimal germination conditions determined by RSM were a temperature of 28 °C and 24 to enhance the contents of riboflavin, FPC and antioxidant activity in moringa sprouts, while 36 °C in combination with times of 24 and 96 h, maximized the levels of thiamine and GABA, respectively (Table 5).

The regression models obtained were further validated by comparing the experimental results with those predicted by the models under the optimal conditions (Table 5). Predicted values closely matched with the experimental ones, confirming the adequacy of the regression models describing the relationship between germination conditions and the studied response variables in germinated moringa.

## 4. Conclusions

This investigation shows that germination is a feasible approach to improve the nutritional and bioactive quality of moringa seeds, but the extent of nutritional and functional improvement depended on the germination temperature and time. Germination enhanced the content of fiber, fat, riboflavin, GABA and FPC, as well as the antioxidant potential of moringa sprouts. Total GLS decreased during germination at 96 h while some individual GLS (glucomoringin and glucotropaeolin) increased during moringa sprouting at selected conditions. Multi-response optimization carried out by RSM allowed to identify the optimal germination conditions that maximize the quality of moringa sprouts, which were 28 °C for 24 h for enhancing the accumulation of riboflavin, free phenolics and antioxidant activity, while the highest thiamine levels were observed at 36 °C for 24 h. The accumulation of GLS and GABA was favored by higher germination temperatures and longer germination times (36 °C for 24 h). These results provide interesting insights for the production of moringa sprouts with high content of nutrients and bioactive compounds and large antioxidant potential that can be consumed as such or used as functional ingredients in novel foodstuffs such as pasta, instant powdered foods, snacks, bakery products and drinks.

## Figures and Tables

**Table 1:** Proximate composition (g/100 g d.m.) of moringa seed and their sprouts obtained at different germination conditions.

Sample	T (°C)	t (h)	Ash	Protein	Lipids	Fiber	Total Carbohydrates
Seed	-	-	3.34 ± 0.00^a^	28.01 ± 0.63^e^	28.73 ± 0.04^cd^	24.41 ± 1.24^d^	35.32 ± 0.83^a^
Sprouts	28	24	2.98 ± 0.03^c^	29.59 ± 0.66^bc^	30.13 ± 0.04^bc^	28.32 ± 1.59^ab^	34.45 ± 0.73^a^
		60	3.14 ± 0.03^b^	30.64 ± 0.50^b^	31.89 ± 0.30^ab^	29.72 ± 2.09^a^	29.25 ± 0.24^bc^
		96	2.99 ± 0.02^c^	28.43 ± 0.97^e^	29.26 ± 1.05^cd^	26.92 ± 0.05^c^	34.79 ± 2.05^a^
	32	24	3.19 ± 0.06^b^	30.04 ± 0.70^bc^	32.08 ± 1.14^ab^	26.12 ± 1.56^bcd^	29.54 ± 1.84^bcd^
		60	3.20 ± 0.40^b^	31.86 ± 0.73^a^	33.39 ± 1.29^a^	26.20 ± 2.37^bcd^	28.00 ± 1.14^cd^
		96	3.17 ± 0.11^b^	32.21 ± 0.46^a^	33.18 ± 0.50^a^	27.11 ± 1.61^abcd^	26.84 ± 1.02^d^
	36	24	2.83 ± 0.05^d^	28.80 ± 0.06^de^	30.40 ± 1.33^bc^	28.01 ± 0.74^ab^	34.04 ± 1.28^a^
		60	3.13 ± 0.02^b^	31.79 ± 0.43^a^	30.27 ± 0.38^bc^	23.97 ± 0.27^d^	30.11 ± 0.42^b^
		96	3.14 ± 0.00^b^	28.99 ± 0.20^cde^	27.87 ± 1.53^d^	27.77 ± 1.75^abc^	35.01 ± 1.31^a^

Data are the mean ± standard deviation of three independent experiments. Different superscript letters within a column indicate significant differences (one-way ANOVA, post hoc Duncan’s test, *p* ≤ 0.05). T: germination temperature; t: germination time; d.m.: dry matter.

**Table 2:** Content of thiamine and riboflavin (μg/100 g d.m.) in moringa seed and their sprouts obtained at different germination conditions.

Sample	T (°C)	t (h)	Thiamine	Riboflavin
Seed	-	-	1113.5 ± 18.8^a^	179.11 ± 6.4^de^
Sprouts	28	24	1090.8 ± 35.3^a^	625.20 ± 52.1^a^
		60	1099.4 ± 5.0^a^	221.27 ± 10.8^b^
		96	1112.0 ± 7.3^a^	215.54 ± 8.4^bc^
	32	24	995.4 ± 8.6^b^	152.80 ± 13.0^d^
		60	1005.7 ± 50.3^b^	153.95 ± 12.5^d^
		96	979.7 ± 8.0^b^	81.50 ± 10.9^f^
	36	24	1113.7 ± 51.9^a^	193.33 ± 17.1^cd^
		60	1090.2 ± 30.3^a^	115.10 ± 13.6^e^
		96	1001.9 ± 27.4^b^	65.40 ± 3.8^g^

Data are the mean ± standard deviation of three independent experiments. Different superscript letters within a column indicate significant differences (one-way ANOVA, post hoc Duncan’s test *p* ≤ 0.05). T: germination temperature; t: germination time.

**Table 3:** Content of glucomoringin, sinalbin, glucotropaeolin and total glucosinolates (GLS) (µmol/g d.m.) in moringa seed and their sprouts obtained at different germination conditions.

Sample	T (°C)	t (h)	Glucomoringin	Sinalbin	Glucotropaeolin	Total GLS
Seed	-	-	197.4 ± 0.2^d^	7.10 ± 0.29^a^	ND^f^	204.5 ± 0.1^c^
Sprouts	28	24	90.0 ± 1.2^g^	4.11 ± 0.05^b^	ND^f^	94.1 ± 1.2^e^
		60	176.7 ± 1.9^e^	3.23 ± 0.46^c^	0.90 ± 0.02^d^	180.8 ± 2.4^d^
		96	205.1 ± 0.9^c^	2.62 ± 0.03^d^	1.28 ± 0.16^c^	209.02 ± 0.8^c^
	32	24	234.9 ± 0.6^a^	2.64 ± 0.02^d^	ND^f^	237.5 ± 0.6^a^
		60	214.5 ± 3.3^b^	2.56 ± 0.08^de^	0.38 ± 0.11^e^	217.5 ± 3.2^b^
		96	170.2 ± 3.5^f^	2.51 ± 0.20^de^	3.69 ± 0.70^b^	176.4 ± 3.0^d^
	36	24	202.4 ± 6.9^cd^	2.47 ± 0.08^de^	ND^f^	204.9 ± 6.9^c^
		60	232.9 ± 6.5^a^	2.58 ± 0.10^de^	0.39 ± 0.01^e^	235.8 ± 6.6^a^
		96	171.4 ± 1.1^ef^	2.23 ± 0.13^e^	4.88 ± 0.31^a^	181.3 ± 5.5^d^

Data are the mean ± standard deviation of four replicates. Different superscript letters within a column indicate significant differences (one-way ANOVA, post hoc Duncan’s test *p* ≤ 0.05). T: germination temperature; t: germination time; ND: not detected.

**Table 4:** Content of GABA (mg/100 g d.m.), FPC (mg GAE/100 g d.m) and antioxidant activity (mg TE/100 g d.m) in moringa seed and their sprouts obtained at different germination conditions.

Sample	T (°C)	t (h)	GABA	FPC	Antioxidant Activity
Seed	-	-	50.1 ± 2.2^f^	418.0 ± 32.0^e^	1531.4 ± 129.1^g^
Sprouts	28	24	97.6 ± 9.6^d^	753.5 ± 61.0^a^	5172.9 ± 522.3^a^
		60	101.8 ± 4.8^d^	646.9 ± 40.2^b^	2199.9 ± 170.9^de^
		96	197.6 ± 19.9^b^	335.7 ± 22.6^f^	1904.7 ± 171.4^f^
	32	24	33.9 ± 3.6^g^	593.5 ± 50.7^c^	2541.9 ± 245.4^b^
		60	129.2 ± 7.8^c^	562.0 ± 46.8^d^	2467.0 ± 237.3^bc^
		96	136.2 ± 20.5^c^	576.1 ± 52.8^cd^	2333.4 ± 196.0^cd^
	36	24	75.0 ± 7.3^e^	340.8 ± 33.3^f^	2124.1 ± 203.1^ef^
		60	107.0 ± 9.3^d^	324.2 ± 21.4^f^	2097.3 ± 133.1^ef^
		96	291.2 ± 19.9^a^	645.7 ± 35.9^f^	1108.1 ± 111.1^h^

Data are the mean ± standard deviation of three independent experiments. Different superscript letters within a column indicate significant differences (one-way ANOVA, post hoc Duncan’s test *p* ≤ 0.05). T: germination temperature; t: germination time; GABA: γ-aminobutyric acid; FPC: free phenolic compounds; GAE: gallic acid equivalents; TE: Trolox equivalents.

**Table 5:** Predictive polynomial equations obtained by RSM and the predicted and experimental values for thiamine, riboflavin, GLS, GABA, FPC contents and antioxidant activity in the optimal germination conditions.

					Optimal Conditions	
Response	Predicted Model	R^2^	T (°C)	t (h)	Predicted Values	Experimental Values
Thiamine	Y(T,t) = 5978.36 − 325.97T + 8.72 t + 5.37T^2^ − 0.29T × t	0.83	36	24	1156.6	1113.7 ± 51.9
Riboflavin	Y(T,t) = 10,034.0 − 536.61T − 26.47 t + 7.41T^2^ + 0.54 T × t + 0.05t^2^	0.83	28	24	577.7	625.2 ± 52.1
GLS	Y(T,t) = 3057.2 − 187.21T + 1.72 t + 2.83T^2^ + 0.07 T × t − 0.03t^2^	0.87	36	96	246.9	178.55 ± 0.6
GABA	Y(T,t) = 3494.6 − 204.17T − 6.84t + 3.01T^2^ + 0.20T × t + 0.02t^2^	0.83	36	96	255.7	291.2 ± 19.9
FPC	Y(T,t) = 846.17 + 91.88T − 48.63t − 2.98T^2^ + 1.35T × t + 0.04t^2^	0.86	28	24	840.4	753.5 ± 61.0
AO	Y(T,t) = 23,028.9 − 755.53T − 178.11t + 5.26T^2^ + 4.25T × t + 0.17t^2^	0.76	28	24	4671	5172 ± 522

GLS: glucosinolates; GABA: γ-aminobutyric acid; FPC: free phenolic compounds; AO: antioxidant activity; T: temperature; t: time; RSM: response surface methodology. All results are expressed in d.m.
